# Engineered variants provide new insight into the structural properties important for activity of the highly dynamic, trimeric protein disulfide isomerase ScsC from *Proteus mirabilis*

**DOI:** 10.1107/S2059798319000081

**Published:** 2019-02-26

**Authors:** Emily J. Furlong, Fabian Kurth, Lakshmanane Premkumar, Andrew E. Whitten, Jennifer L. Martin

**Affiliations:** a Institute for Molecular Bioscience, The University of Queensland, St Lucia, QLD 4072, Australia; b Griffith Institute for Drug Discovery, Griffith University, Nathan, QLD 4111, Australia; c Australian Nuclear Science and Technology Organisation, Lucas Heights, NSW 2234, Australia

**Keywords:** suppressor of copper sensitivity protein C, ScsC, disulfide isomerases, copper tolerance, *Proteus mirabilis*

## Abstract

Suppressor of copper sensitivity protein C from *Proteus mirabilis* (PmScsC) is a homotrimeric disulfide isomerase that plays a role in copper tolerance, which is a key virulence trait of this uropathogen. Each protomer of the enzyme has an N-terminal trimerization stem (59 residues) containing a flexible linker (11 residues) connected to a thioredoxin-fold-containing catalytic domain (163 residues). Here, two PmScsC variants, PmScsCΔN and PmScsCΔLinker, are characterized. PmScsCΔN is an N-terminally truncated form of the protomer with two helices of the trimerization stem removed, generating a protein with dithiol oxidase rather than disulfide isomerase activity. The crystal structure of PmScsCΔN reported here reveals, as expected, a monomer that is structurally similar to the catalytic domain of native PmScsC. The second variant, PmScsCΔLinker, was designed to remove the 11-amino-acid linker, and it is shown that it generates a protein that has neither disulfide isomerase nor dithiol oxidase activity. The crystal structure of PmScsCΔLinker reveals a trimeric arrangement, with the catalytic domains packed together very closely. Small-angle X-ray scattering analysis found that native PmScsC is predominantly trimeric in solution even at low concentrations, whereas PmScsCΔLinker exists as an equilibrium between monomeric, dimeric and trimeric states, with the monomeric form dominating at low concentrations. These findings increase the understanding of disulfide isomerase activity, showing how (i) oligomerization, (ii) the spacing between and (iii) the dynamic motion of catalytic domains in PmScsC all contribute to its native function.

## Abstract 2

The structure and function of a bacterial trimeric protein disulfide isomerase was investigated by characterising two variants in which key structural regions were deleted. In one case the effect on structure and function was predictable, while in the other we found unintended consequences for both structure and function.

## 1. Introduction   

Protein disulfide isomerases are enzymes that proofread and shuffle incorrect disulfide bonds in misfolded protein substrates, and are important for the correct folding and function of many secreted proteins (Berkmen *et al.*, 2005 ▸; Hiniker & Bardwell, 2004 ▸). The prototypical bacterial di­sulfide isomerase is disulfide-bond (Dsb) protein C from *Escherichia coli* (EcDsbC; Zapun *et al.*, 1995 ▸; Missiakas *et al.*, 1994 ▸; Shevchik *et al.*, 1994 ▸). EcDsbC functions as a dimer; when the N-terminal dimerization domain is deleted the resulting protein lacks disulfide isomerase activity (Sun & Wang, 2000 ▸) and is also unable to interact with its redox partner EcDsbD (Goldstone *et al.*, 2001 ▸). Each EcDsbC protomer has a thioredoxin-fold catalytic domain with a redox-active motif consisting of two cysteines separated by two other amino acids (C*XX*C; McCarthy *et al.*, 2000 ▸). The disulfide-isomerase activity of EcDsbC requires that the catalytic cysteines are in the dithiol-reduced form (Darby *et al.*, 1998 ▸), and this form is generated by the interaction of EcDsbC with its redox-partner membrane protein EcDsbD (Goldstone *et al.*, 2001 ▸).

Recently, we reported the structural and functional characterization of *Proteus mirabilis* suppressor of copper sensitivity protein C (PmScsC; Furlong *et al.*, 2017 ▸). Like DsbC, PmScsC is a protein disulfide isomerase with a thioredoxin-fold catalytic domain (Furlong *et al.*, 2017 ▸), redox-active cysteines (Furlong *et al.*, 2017 ▸) and a redox-partner membrane protein (PmScsB) that reduces the active-site cysteines (Furlong *et al.*, 2018 ▸). Unlike DsbC, PmScsC is trimeric rather than dimeric (Furlong *et al.*, 2017 ▸). Three crystal structures and small-angle X-ray scattering (SAXS) analysis also revealed that PmScsC is highly dynamic (Furlong *et al.*, 2017 ▸). The region responsible for this conformational flexibility is an 11-amino-acid linker within the trimerization stem of the protein. Replacement of the flexible linker with a rigid helical peptide linker (PmScsC RHP) limits the rigid-body motion of the catalytic domain relative to the trimer stalk, and generates a protein that is inactive as a disulfide isomerase. Moreover, PmScsC lacking the trimerization stem (the N-terminal 41 residues; PmScsCΔN) is also inactive as a disulfide isomerase but does have dithiol oxidase activity (Furlong *et al.*, 2017 ▸). Neither the native protein nor the PmScsC RHP variant exhibited dithiol oxidase activity. The difference in the activity profile between the native protein (disulfide isomerase) and PmScsCΔN (dithiol oxidase) was proposed to be a consequence of their different oligomeric states.

**Figure 1:**
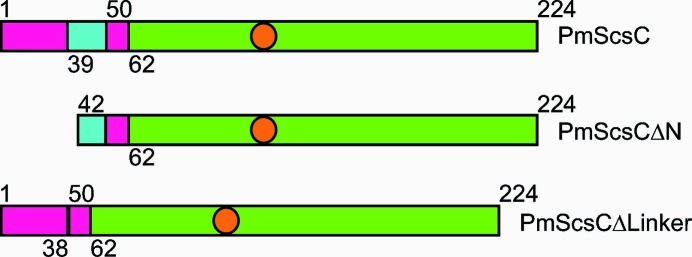
Schematic representation of the PmScsC variants used in this study. The region corresponding to the catalytic domain is shown in green, the trimerization stem is coloured magenta and the region representing the flexible linker is shown in cyan. The orange circles represent the approximate positions of the redox-active cysteines.

Here, we report the structural characterization of PmScsCΔN and the structural and functional characterization of PmScsCΔLinker (a variant in which the 11-amino-acid flexible linker is deleted; Fig. 1 ▸). The crystal structure of PmScsCΔN reveals, as expected, a monomeric protein that closely resembles the catalytic domain from the full-length PmScsC protomer. We show that the PmScsCΔLinker variant lacks both disulfide isomerase and dithiol oxidase activities. ­The crystal structure reveals a trimeric arrangement for PmScsCΔLinker, with the catalytic domains closely packed against each other. Conversely, SAXS analysis indicates that the oligomeric state of PmScsCΔLinker is concentration-dependent, with a monomeric form present at low concentrations and a dimeric form dominating at higher concentrations. For comparison, we analysed a concentration series of full-length native PmScsC by SAXS, showing that the dominant species of the native protein in solution is trimeric even at very low concentrations.

## 2. Materials and methods   

### 2.1. Molecular biology, protein expression and purification   

The constructs for the expression of PmScsC and PmScsCΔN were created previously (Furlong *et al.*, 2017 ▸) using UniProt sequence B4EV21 without the predicted periplasmic signalling sequence (*i.e.* residues 22–243). To make the PmScsCΔLinker expression construct, the DNA encoding the flexible peptide linker (residues 39-KADEQQAQFRQ-49) in PmScsC was deleted from the *P. mirabilis* *scsC* gene using overlap extension PCR with the primers 5′-GCAATCATGGCTCTGCAGACGAAAGCACTGGCTAGCGAACATGATGCC-3′ and 5′-GGCATCATGTTCGCTAGCCAGTGCTTTCGTCTGCAGAGCCATGATTGC-3′. Ligation-independent cloning was then used to insert the mutated gene into pMCSG7. Sequencing of the construct confirmed that the encoded protein was in-frame with an N-terminal His_6_ tag and TEV protease cleavage site, and revealed that residue 6 of PmScsCΔLinker was also mutated from asparagine to lysine. The protein residues are numbered based on the sequence of full-length PmScsC after TEV protease cleavage (starting S_1_N_2_A_3_…). All proteins were expressed in *Escherichia coli* BL21 (DE3) pLysS cells and purified using immobilized metal-affinity chromatography, TEV protease cleavage and size-exclusion chromatography as described previously (Furlong *et al.*, 2017 ▸). SDS–PAGE with Coomassie staining was used to estimate that the purity of each protein was >95%. The concentration of the proteins was determined by measuring the *A*_280_ of the samples and adjusting for the theoretical *A*_280_ of a 1 mg ml^−1^ solution of the protein [Abs 0.1%(*w*/*v*)]. The theoretical *A*_280_ values for 0.1%(*w*/*v*) determined from *ProtParam* (Gasteiger *et al.*, 2005 ▸) were 0.528, 0.643 and 0.558 for PmScsC, PmScsCΔN and PmScsCΔLinker, respectively.

### 2.2. Disulfide isomerase and dithiol oxidase assays   

The disulfide isomerase and dithiol oxidase activities of PmScsCΔLinker were determined as described previously (Furlong *et al.*, 2017 ▸). Briefly, to determine the disulfide isomerase activity, the ability of the protein to refold scrambled RNase A (scRNase A) was assessed. 0.23 mg ml^−1^ (10 µ*M*) PmScsCΔLinker was incubated with 40 µ*M* scRNase A; samples were taken at various time points and the RNase A activity was measured in a spectrophotometric assay with a final concentration of 3 m*M* cytidine 3′,5′-cyclic monophos­phate (cCMP). Results are reported as a percentage of the activity of the RNase A-only control and are adjusted for the scRNase A-only negative control. The dithiol oxidase activity of PmScsCΔLinker was assessed by measuring the ability of the protein to form a disulfide bond between the two cysteines in a synthetic peptide over time. The peptide substrate has 1,4,7,10-tetraazacyclododecane-1,4,7,10-tetraacetic acid (DOTA) bound to europium at the N-terminus and lysine methoxycoumarin amide (MCA) at the C-terminus. As the cysteines become oxidized, the termini of the peptide are brought into close proximity, which causes an increase in fluorescence. PmScsCΔLinker (80 n*M* or 0.002 mg ml^−1^) was incubated with 8 µ*M* of the peptide substrate and the increase in fluorescence over time was monitored using a Synergy H1 Hybrid plate reader (BioTek, Vermont, USA), reading fluorescence at excitation and emission wavelengths of 340 and 615 nm, respectively. The rate of peptide oxidation is reported relative to the activity of the prototypical dithiol oxidase EcDsbA. The disulfide isomerase and dithiol oxidase assays were repeated two and three times, respectively, and the mean and standard deviation for each is reported.

### 2.3. Crystallization   

Initial crystallization hits for both PmScsCΔN and PmScsCΔLinker were identified in 96-well hanging-drop vapour-diffusion experiments using commercial crystallization screens set up with a Mosquito robot (TTP Labtech, Melbourn, England) at the University of Queensland Remote Operation Crystallization and X-ray (UQ ROCX) Diffraction Facility. All crystallization experiments were incubated at 293 K and monitored using a Rock Imager with *Rock Maker* software (Formulatrix, Waltham, Massachusetts, USA).

Well diffracting, rod-shaped crystals of PmScsCΔN were obtained in a 96-well screening plate in a drop consisting of 200 nl PEGRx HT (Hampton Research, Aliso Viejo, California, USA) condition C10 [0.1 *M* sodium acetate trihydrate pH 4.5, 30%(*w*/*v*) PEG monomethyl ether 5000] and 200 nl 38 mg ml^−1^ PmScsCΔN in 10 m*M* HEPES pH 7.4. Perfluoropolyether (Hampton Research, Aliso Viejo, California, USA) was used to cryoprotect the crystals before they were flash-cooled in liquid nitrogen.

Needle-like crystals of PmScsCΔLinker were obtained in well F12 of the commercial screen JCSG-*plus* (Molecular Dimensions, Newmarket, England) and were then optimized in a 24-well plate grid screen around the original condition. This optimization resulted in more robust and well diffracting crystals. Data were collected from a crystal grown using 1 µl 0.1 *M* HEPES pH 7, 32%(*v*/*v*) Jeffamine M-600 and 1 µl of 40 mg ml^−1^ PmScsCΔLinker in 10 m*M* HEPES pH 7.4, 150 m*M* NaCl. The crystal was cryoprotected in the crystallization condition plus 20% ethylene glycol before being flash-cooled in a cryostream (100 K).

### 2.4. Data collection, structure solution and refinement   

Data for the PmScsCΔN structure were collected using a wavelength of 0.95370 Å at 100 K on the MX2 beamline at the Australian Synchrotron using the *Blu-Ice* software (McPhillips *et al.*, 2002 ▸). The data were processed and scaled in the trigonal space group *P*312 using *XDS* (Kabsch, 2010 ▸), *POINTLESS* (Evans, 2006 ▸) and *SCALA* (Evans, 2006 ▸). Phases were obtained by molecular replacement in *Phaser* (McCoy *et al.*, 2007 ▸) using *Salmonella enterica* serovar Typhimurium ScsC (PDB entry 4gxz; Shepherd *et al.*, 2013 ▸) as the model. The initial model was improved by iterative model building in *Coot* (Emsley *et al.*, 2010 ▸) and refinement in *PHENIX* (Adams *et al.*, 2010 ▸). The quality of the final PmScsCΔN model was assessed using *MolProbity* (Chen *et al.*, 2010 ▸) and had an *R*_free_ value of 20.8% and an *R*_work_ value of 17.5% at a resolution of 2.15 Å. The coordinates and structure factors have been deposited in the Protein Data Bank under the code 6nen, and raw data images are available from https://doi.org/10.14264/uql.2018.843.

Data for the PmScsCΔLinker structure were collected at UQ ROCX under cryogenic conditions (100 K) using a Rigaku FR-E SuperBright X-ray generator, producing X-rays at a wavelength of 1.54187 Å, and a Rigaku Saturn 944 CCD area detector. The data collection was performed with the Rigaku *CrystalClear* (v.2.0) program. Data were processed in *iMosflm* (v.7.1.3; Battye *et al.*, 2011 ▸) with space group *H*32 (also known as *R*32:*h*) and were scaled in *AIMLESS* (Evans & Murshudov, 2013 ▸) as implemented in the *CCP*4 suite (v.6.5.008; Winn *et al.*, 2011 ▸). Owing to detector-distance constraints at UQ ROCX, diffraction data were limited to a highest resolution of 2.08 Å. The crystals diffracted strongly, resulting in very high mean *I*/σ(*I*) values (27.2 overall and 11.8 in the highest resolution shell). The structure was phased by molecular replacement using *Phaser* (McCoy *et al.*, 2007 ▸) and the catalytic domain of the compact PmScsC crystal structure (residues 47–224; PDB entry 4xvw) was used as the search model. The trimerization domain (without the 11-amino-acid flexible linker) was then manually built into the electron density in *Coot* (Emsley *et al.*, 2010 ▸). The structure was refined using rounds of *phenix.refine* and manual adjustment in *Coot*, with reference to *MolProbity* (Chen *et al.*, 2010 ▸). The final *R*_free_ and *R*_work_ values are 21.0% and 17.4%, respectively, at a resolution of 2.08 Å. The coordinates and structure factors have been deposited in the Protein Data Bank under the code 6mhh, and raw data images are available from https://doi.org/10.14264/uql.2018.842. All structural figures were produced in *PyMOL* (v.1.6; Schödinger) and structural alignments were performed in *Coot* using the ‘superpose’ command (Emsley *et al.*, 2010 ▸) which aligns C^α^ atoms by least squares. The *CONTACT* program from the *CCP*4 suite (Winn *et al.*, 2011 ▸) was used to analyse the interactions between the trimerization stems of PmScsC and PmScsCΔLinker. Data-collection and refinement statistics are given in Table 1 ▸.

### 2.5. SAXS   

SAXS data were collected on the SAXS/WAXS beamline at the Australian Synchrotron (Kirby *et al.*, 2013 ▸). Data reduction was carried out using the *scatterBrain* software (v.2.71; Australian Synchrotron; http://archive.synchrotron.org.au/aussyncbeamlines/saxswaxs/software-saxswaxs) and the data were corrected for solvent scattering, sample transmission and detector sensitivity. The 0.1, 0.5, 2.0, 5.0 and 10.0 mg ml^−1^ samples of PmScsCΔN were made by diluting a 30 mg ml^−1^ stock in 10 m*M* HEPES pH 7.4, 150 m*M* NaCl. For PmScsC and PmScsCΔLinker, samples were taken throughout centrifugal concentration and the concentration of these samples (based on *A*_280_ readings) was adjusted to 0.1, 0.5, 2.0, 5.0 or 10.0 mg ml^−1^ in 10 m*M* HEPES pH 7.4, 150 m*M* NaCl. These samples, along with the blank (10 m*M* HEPES pH 7.4, 150 m*M* NaCl), were loaded into a 96-well plate (Table 2 ▸; Trewhella *et al.*, 2017 ▸). The estimated molecular mass was calculated from the Porod volume (Fischer *et al.*, 2010 ▸) and from *I*(0) using contrast and partial specific volumes determined from the protein sequences using *MULCh* (v.1.1; Whitten *et al.*, 2008 ▸). For PmScsCΔN, data processing and Guinier analysis was performed using *PRIMUS* (v.3.2; Konarev *et al.*, 2003 ▸). The pair-distance distribution function [*p*(*r*)] was generated from the experimental data using *GNOM* (v.4.6; Svergun, 1992 ▸), from which *I*(0), *R*_g_ and *D*_max_ were determined. *DAMMIN* (v.5.3; Svergun, 1999 ▸) was used to generate 16 dummy-atom models for the protein, which were averaged using *DAMAVER* (v.2.8.0; Volkov & Svergun, 2003 ▸), and the resolution of the averaged structures was estimated using *SASRES* (Tuukkanen *et al.*, 2016 ▸). All 16 dummy-atom models were used in the averaging procedure. The model scattering curve for PmScsCΔN (SASBDB ID SASDEK4) was calculated from the crystal structure (PDB entry 6nen) using *CRYSOL* (v.2.8.3; Svergun *et al.*, 1995 ▸).

For PmScsC (SASBDB ID SASDER4) and PmScsCΔLinker (SASBDB ID SASDEQ4), the data were modelled as a linear combination of model scattering curves to quantify the proportions of different oligomeric states in solution (Hu *et al.*, 2012 ▸). Structures of the PmScsC monomer (chain *A*), dimer (chains *A* and *B*) and trimer (chains *A*, *B* and *C*) were taken from a *CORAL* model previously optimized against the SAXS data for PmScsC (SASBDB ID SASDB94; Furlong *et al.*, 2017 ▸). Structures of the PmScsCΔLinker monomer (chain *A*), dimer (chains *A* and *B*) and trimer (chains *A*, *B* and *C*) were taken from the crystal structure of PmScsCΔLinker (PDB entry 6mhh). Scattering curves for each oligomer were calculated using *CRYSOL* (v.2.8.3; Svergun *et al.*, 1995 ▸). The calculated scattering curves (with units of e^2^) were multiplied by *r*_e_^2^ (cm^2^ e^−2^) × *N*_A_ (mol^−1^) × 10^−3^ (l cm^−3^) = 4.78181 × 10^−5^ (cm^−1^ e^−2^ mol^−1^ l). This normalization permits the coefficients arising from a fit to the experimental data on an absolute scale to be interpreted as the molar concentration of each oligomer present in solution. For PmScsC, a linear combination of monomer and trimer scattering curves was fitted to the scattering data at each concentration. Additionally, to reduce the number of free parameters, the concentration of monomer for each SAXS curve was optimized together with a common equilibrium constant, *K*_t_ (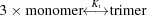), and the concentration of trimer was calculated from *K*_t_ and the momomer concentration. For PmScsCΔLinker, a linear combination of monomer, dimer and trimer scattering curves was fitted to the scattering data at each concentration. Again, to reduce the number of free parameters the concentration of monomer for each SAXS curve was also optimized together with two common equilibrium constants, *K*_d_ (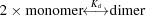), and *K*_t_ (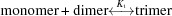). The concentration of dimer was calculated from *K*_d_ and the monomer concentration, while the concentration of trimer was calculated from *K*_t_ and the concentrations of monomer and dimer. From the model concentrations of each component, a total protein concentration was also calculated for each SAXS data set and these are reported in Table 3 ▸. These calculated concentrations are systematically lower than the measured protein concentrations.

## 3. Results   

### 3.1. PmScsCΔN crystal structure and SAXS analysis   

**Figure 2:**
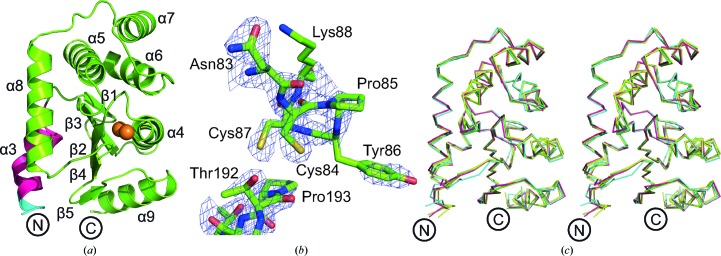
Structural characterization of PmScsCΔN. (*a*) Structure of the PmScsCΔN variant. Structural features are coloured as in Fig. 1 ▸ and the secondary-structure elements are labelled based on the native PmScsC structures (Furlong *et al.*, 2017 ▸). (*b*) Electron density of the catalytic region in PmScsCΔN. The wire mesh represents the *F*_o_ − *F*_c_ electron-density difference map (generated in *phenix.refine* with the region of interest omitted from the structure) contoured to 3σ and shown within a 2 Å radius of each atom. (*c*) Stereo diagram of the structural alignment of PmScsCΔN (green) with residues 46–221 of chain *A* from the compact (cyan; PDB entry 4xvw), transitional (yellow; PDB entry 5idr) and extended (magenta; PDB entry 5id4) PmScsC structures (Furlong *et al.*, 2017 ▸; r.m.s.d. of 0.51–0.68 Å, 174 residues aligned). This superimposition shows that the structures are similar whether or not the trimerization domain is present.

**Figure 3:**
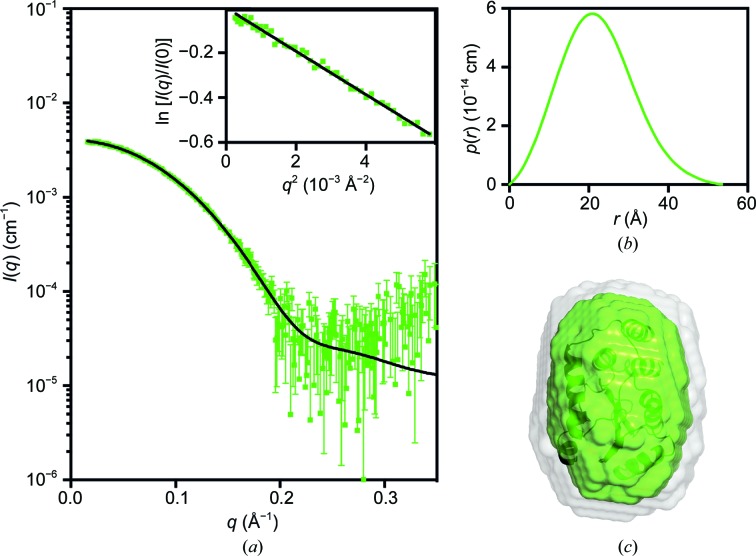
Small-angle X-ray scattering data for PmScsCΔN. (*a*) Measured scattering data for PmScsCΔN. The scattering profile of a rigid-body model is shown as a solid black line overlaid on the scattering data for PmScsCΔN [χ^2^ = 1.12; CorMap test (Franke *et al.*, 2015 ▸), 302 points, *C* = 14, *P* = 0.018]. Inset: Guinier plot for PmScsCΔN (*R* ^2^ = 0.991). (*b*) The pair-distance distribution function, *p*(*r*), derived from the scattering data is indicative of a globular structure for PmScsCΔN (multiplied by a factor of four for clarity) with a maximum dimension of ∼55 Å. (*c*) Probable shape of PmScsCΔN obtained from the filtered average of 16 dummy-atom models (green envelope): χ^2^ = 1.079 ± 0.001; NSD = 0.522 ± 0.007; resolution = 19 ± 2 Å. The image in (*c*) was generated using *PyMOL*, where the grey shape represents the total volume encompassed by the aligned dummy-atom models and the crystal structure is shown as a ribbon diagram aligned with the filtered model (dark green).

The design and biochemical characterization of PmScsCΔN has previously been reported (Furlong *et al.*, 2017 ▸). The present work reports the crystal structure of this variant. Crystals of the PmScsCΔN mutant diffracted to a resolution of 2.15 Å at the Australian Synchrotron. The crystal structure of the mutant was solved using molecular replacement and there was only one PmScsCΔN molecule in the asymmetric unit (Table 1 ▸, Figs. 2 ▸ *a* and 2 ▸ *b*). In the crystal packing, PmScsCΔN makes contact with six symmetry-related molecules, but none involve packing against the catalytic motif or the positively charged surface patch that surrounds it. The structure of PmScsCΔN is very similar to the structure of the catalytic domain present in each of the three previously reported PmScsC structures (r.m.s.d. of 0.51–0.68 Å for the C^α^ atoms of 174 residues aligned between chain *A* of each structure; Fig. 2 ▸ *c*). Small-angle scattering (Table 2 ▸) was used to determine the low-resolution solution structure of PmScsCΔN (Fig. 3 ▸). The scattering data for PmScsCΔN are representative of a monomeric globular particle and are consistent with the scattering curve calculated from the crystal structure of PmScsCΔN (PDB entry 6nen). The crystal structure also shows good agreement with the dummy-atom model derived from the scattering data (Fig. 3 ▸ *c*).

### 3.2. Activity of PmScsCΔLinker   

**Figure 4:**
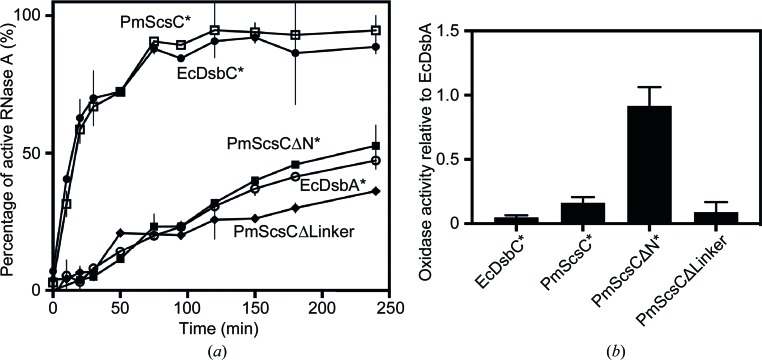
Biochemical activity of PmScsCΔLinker. (*a*) The ability of PmScsCΔLinker to isomerize the incorrect disulfide bonds in scrambled RNase A is poor; it is even lower than that of the dithiol oxidase EcDsbA. (*b*) PmScsCΔLinker has poor dithiol oxidase activity; it is much lower than that of monomeric PmScsCΔN and similar to those of the protein disulfide isomerases EcDsbC and PmScsC. Asterisks indicate data published previously (Furlong *et al.*, 2017 ▸). Error bars in both panels represent the standard deviation of the mean.

A key structural feature of native PmScsC is an 11-amino-acid flexible linker that is situated in the trimerization stem and links to the catalytic domain. This short region facilitates the conformational variations observed in the crystal structures and the dynamic motion of the trimeric protein observed in SAXS analyses (Furlong *et al.*, 2017 ▸). To better understand the role of the flexible linker, we designed a PmScsCΔLinker variant in which the 11-residue linker is deleted and characterized its function in standard assays. We first assessed the activity of PmScsCΔLinker in the scrambled RNase A assay and found that it was poorly active, with baseline activity similar to that of a typical dithiol oxidase such as EcDsbA or PmScsCΔN (Fig. 4 ▸ *a*). We then assessed its activity in the model peptide dithiol oxidase assay, in which EcDsbA and PmScsCΔN are both active. However, unlike the monomeric proteins EcDsbA and PmScsCΔN, PmScsCΔLinker was also inactive in the dithiol oxidase assay (Fig. 4 ▸ *b*). Disulfide isomerase activity is thought to be associated with the oligomerization of thioredoxin-fold proteins (Furlong *et al.*, 2017 ▸; Ke *et al.*, 2006 ▸; Sun & Wang, 2000 ▸), so we were interested to determine whether PmScsCΔLinker forms a trimer, like the full-length native protein, or a monomer, like the truncated PmScsCΔN variant.

### 3.3. The crystal structure reveals a trimeric form of PmScsCΔLinker   

**Figure 5:**
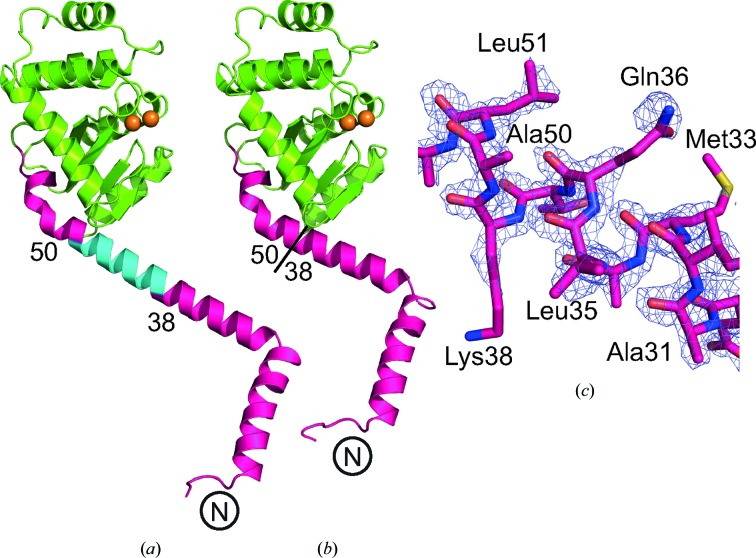
Crystal structure of PmScsCΔLinker. (*a*) Single protomer of the extended PmScsC (PDB entry 5id4). (*b*) Single protomer of PmScsCΔLinker. The different features of the crystal structures are coloured as in Fig. 1 ▸. The N-terminus of each structure is labelled and the positions of the residues flanking the flexible linker in the native structure are indicated. (*c*) Electron density around the deleted region in the PmScsCΔLinker structure. The wire mesh represents the *F*_o_ − *F*_c_ electron-density difference map (generated in *phenix.refine* with the region of interest omitted from the structure) contoured to 3σ and shown within a 2 Å radius of each atom.

The three crystal structures of native PmScsC reported previously show that the linker residues can adopt helical (extended-conformation), strand (intermediate-conformation) or loop (compact-conformation) secondary structures (Furlong *et al.*, 2017 ▸). In the extended-conformation crystal structure the linker adopts a helical conformation, thereby creating a very long helix (35 residues, approximately 9.5 turns; Fig. 5 ▸ *a*) extending from the trimerization stalk through into the catalytic domain of PmScsC.

**Figure 6:**
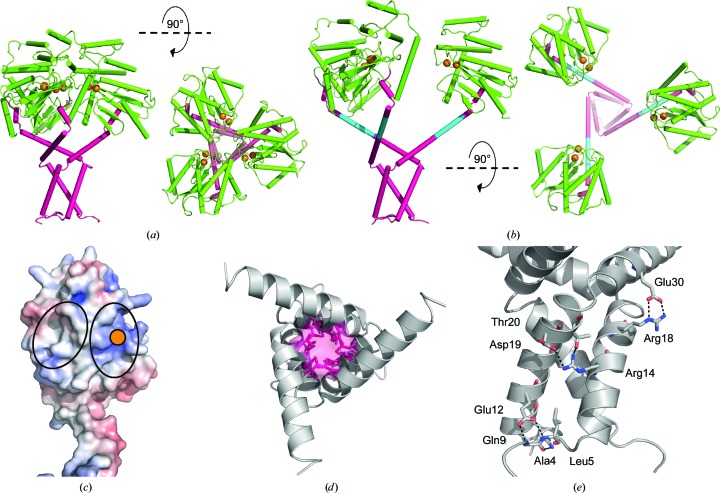
Trimerization of PmScsCΔLinker. (*a*) Side and top views of the catalytic domain in the crystal structure of PmScsCΔLinker. The crystal structure reveals a trimer through crystallographic symmetry. In many respects this crystal structure resembles that of native extended PmScsC shown in (*b*) (PDB entry 5id4). However, the extended helix linking the trimerization domain to the catalytic domain is shorter in PmScsCΔLinker owing to deletion of the linker [cyan in (*b*)]. In both (*a*) and (*b*) the proteins are coloured using the scheme used in Fig. 1 ▸. (*c*) The electrostatic surface potential of the catalytic domain of the PmScsCΔLinker protomer structure. Electrostatic calculations were performed using *APBS* (Jurrus *et al.*, 2018 ▸) and were contoured at −7.5 *kT* e^−1^ (red) and +7.5 *kT* e^−1^ (blue). A solid orange circle indicates the position of the catalytic cysteines; black ovals surround the regions of the catalytic domains in close contact with adjacent catalytic domains in the PmScsCΔLinker crystal structure. (*d*) The hydrophobic core of the trimerization stem of PmScsCΔLinker; side chains of residues contributing to the hydrophobic core are coloured pink. (*e*) Amino-acid residues mediating side-chain polar and electrostatic interactions between the trimerization stems of two PmScsCΔLinker protomers. Black dashed lines represent hydrogen bonds and the residues involved are labelled.

We solved the crystal structure of PmScsCΔLinker at a resolution of 2.08 Å using molecular replacement (Table 1 ▸, Figs. 5 ▸ *b* and 5 ▸ *c*). The variant crystallized in the same space group (*H*32) and crystallization conditions as the extended-conformation PmScsC (PDB entry 5id4; Furlong *et al.*, 2017 ▸). However, the unit-cell dimensions of PmScsCΔLinker are shorter than those of the extended PmScsC structure (*a* = *b* = 64.0, *c* = 299.8 Å compared with *a* = *b* = 86.7, *c* = 330.0 Å). The crystal structure of PmScsCΔLinker, like the crystal structure of the extended form of PmScsC, is trimeric through crystallo­graphic symmetry, with a single protomer in the asymmetric unit (Fig. 6 ▸ *a*). The regions on either side of the linker region reported to be helical in the three native PmScsC crystal structures are directly connected in PmScsCΔLinker and are also helical.

The crystal structure of PmScsCΔLinker is similar to that of extended PmScsC except for a shortening of the long helix by almost three turns (Figs. 5 ▸ *a* and 5 ▸ *b*). This shortened helix in the crystal trimer of PmScsCΔLinker brings the three catalytic domains into much closer proximity in the variant compared with the native extended conformation. This proximity is evident from the distance between the catalytic cysteines of the protomers: 15 Å in PmScsCΔLinker and 34 Å in the extended native structure (measured between the C^β^ atoms of Cys84; Figs. 6 ▸ *a* and 6 ▸ *b*).

The PmScsCΔLinker crystal structure also reveals interactions between each of the catalytic domains of the trimer. The surface interface is formed between a positively charged patch close to the C*XX*C motif on the catalytic domain of one protomer and a neutral/hydrophobic patch on another (Fig. 6 ▸ *c*). This interface is not formed in the extended PmScsC structure, which has both of these regions exposed to solvent, and it is also not formed by crystal packing in the PmScsCΔN structure.

The trimerization stems of all three native PmScsC crystal structures are α-helical with a hydrophobic core and with hydrogen bonds between surface-exposed polar/charged residues. These features are conserved in the crystal structure of PmScsCΔLinker (Figs. 6 ▸ *d* and 6 ▸ *e*). However, it should be noted that PmScsCΔLinker has an additional unanticipated mutation (Asn6Lys) at the N-terminus. In the three native crystal structures the side chain of the Asn6 residue forms an intramolecular hydrogen bond to the main chain of Gln9. In the PmScsCΔLinker crystal structure the side chain of Gln9 adopts the same conformation as in all three native PmScsC structures, but the mutated residue Lys6 does not form an equivalent hydrogen bond to the main chain of Gln9. The lack of this specific hydrogen bond could potentially increase the N-terminal disorder, although the crystal structure suggests there is little impact on the hydrogen bonds and hydrophobic contacts that contribute to trimerization.

### 3.4. SAXS reveals that the oligomeric state of PmScsCΔLinker is concentration dependent   

We had previously observed that the PmScsC RHP variant (in which the flexible linker is replaced by a rigid helix) forms a trimer, like the native protein, but is inactive in the protein disulfide isomerase assay (Furlong *et al.*, 2017 ▸). This lack of activity was thought to be a consequence of the reduced catalytic domain motion observed in solution using small-angle scattering (Furlong *et al.*, 2017 ▸). This finding led to the hypothesis that potent protein disulfide isomerase activity requires at least two catalytic domains (for example a dimer or trimer) which have the ability to move independently of each other (Furlong *et al.*, 2017 ▸). Our present results show that PmScsCΔLinker also forms a trimer and is also inactive as a protein disulfide isomerase. We therefore anticipated that the lack of protein disulfide isomerase activity was a consequence of reduced rigid-body motion of the catalytic domain, and sought to confirm this in solution using small-angle scattering.

**Figure 7:**
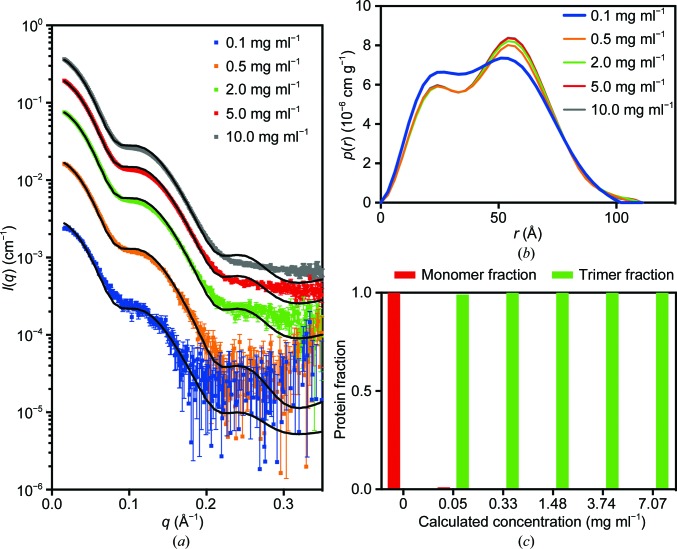
SAXS data for native PmScsC. (*a*) Scattering data collected at ∼0.1 mg ml^−1^ (blue; χ^2^ = 2.55), ∼0.5 mg ml^−1^ (orange; χ^2^ = 7.05), ∼2.0 mg ml^−1^ (green; χ^2^ = 72.85), ∼5.0 mg ml^−1^ (red; χ^2^ = 371.94) and ∼10.0 mg ml^−1^ (grey; χ^2^ = 815.23). The overlaid line (black) on each scattering curve is the fitted linear combination of monomer and trimer scattering curves. (*b*) The pair-distance distribution function, *p*(*r*), derived from the scattering data normalized by concentration shows that the 0.1 mg ml^−1^ data set differs slightly from those at the other concentrations (which all overlay), indicating the possibility that monomer is present in solution at the lowest concentration. (*c*) The fraction of protein present as monomer and trimer, derived from the fitted global optimization models, shows that at total protein concentrations above 0.05 mg ml^−1^ (calculated concentration, or 0.1 mg ml^−1^ measured concentration) the protein is almost exclusively present as a trimer in solution.

**Figure 8:**
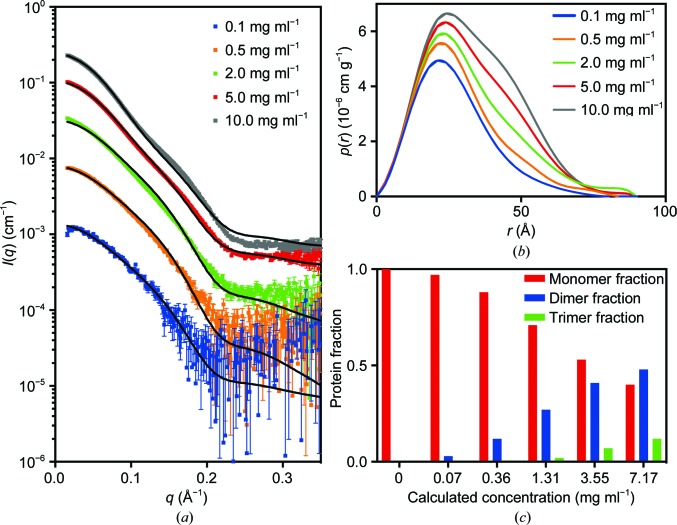
SAXS data for PmScsCΔLinker. (*a*) Scattering data collected at ∼0.1 mg ml^−1^ (blue; χ^2^ = 1.27), ∼0.5 mg ml^−1^ (orange; χ^2^ = 2.19), ∼2.0 mg ml^−1^ (green; χ^2^ = 6.89), ∼5.0 mg ml^−1^ (red; χ^2^ = 37.59) and ∼10.0 mg ml^−1^ (grey; χ^2^ = 177.29). The overlaid line (black) on each scattering curve is the fitted linear combination of monomer and trimer scattering curves. (*b*) The pair-distance distribution function, *p*(*r*), derived from the scattering data normalized by concentration shows that the oligomeric composition of the solution is highly dependent on concentration. (*c*) The fraction of protein present as monomer, dimer and trimer, derived from the fitted global optimization models, shows that the monomeric species is dominant in solution at low concentrations. The concentration of trimer rises slowly and and is predicted to reach a protein fraction of ∼0.2 at 38 mg ml^−1^, which is the concentration at which crystallization was performed.

Unexpectedly, the initial SAXS data for PmScsCΔLinker displayed a strong dependence on concentration, consistent with an equilibrium between different oligomeric forms of PmScsCΔLinker (monomer, dimer and trimer). This result raised the possibility that at low concentrations the wild-type protein may also be present as a mixture of monomers, dimers and trimers, and this might also impact on its function or its regulation. To investigate this possibility further, SAXS data were collected from native PmScsC (Fig. 7 ▸) and PmScsCΔLinker (Fig. 8 ▸) at concentrations between 0.1 and 10.0 mg ml^−1^ (Table 2 ▸). At the lowest concentration (0.1 mg ml^−1^) the shape of the *p*(*r*) function for native PmScsC differed from the shapes of those at higher concentrations, indicating some dissociation of the wild-type protein. A number of approaches were trialled for the analysis, but fitting a linear combination of monomer and trimer scattering to the data at each concentration was found to perform best (Fig. 7 ▸ and Table 3 ▸). The results presented in Table 3 ▸ show generally good agreement for the two modelling approaches that were taken (global fit of a single equilibrium constant to all data sets and fitting of each data set independently). However, it can be seen that there is some disagreement at low concentrations, where the global fit predicts a much lower proportion of monomer in solution than does the independent fitting. Thus, it appears that the higher concentration data sets with higher signal-to-noise ratios may have a disproportionate impact on the magnitude of the determined equilibrium constant. Nonetheless, both methods of analysis show that the bulk of the material is present as the trimeric form at all concentrations. For PmScsCΔLinker, the results are very different; the shape of the *p*(*r*) curve changes significantly with each concentration evaluated. Again, we trialled a number of approaches to fit these data, but fitting a linear combination of monomer, dimer and trimer scattering to the data at each concentration was found to perform best (Fig. 8 ▸ and Table 3 ▸). While the analysis worked well, it is limited by the fact that the structures of the monomer and dimer forms in solution are not known. In each case, we made a simplistic assumption that a monomer adopted the structure of a single protomer from the corresponding crystal structure and a dimer could be represented by removing a single protomer from the trimer crystal structure. We also know that native PmScsC is dynamic in solution (Furlong *et al.*, 2017 ▸). While the model curves provide a reasonable fit to the scattering data, there are systematic differences between the experimental data and the model, especially at higher concentrations. The limitations of the modelling (lack of accurate models, dynamic motion) explain most of this variation.

Overall, the SAXS analysis suggests that only 1–21% of native PmScsC is present as monomers in solution at the lowest concentration of 0.1 mg ml^−1^; we conclude that the trimer is the dominant form of the native protein in solution even at low concentrations. For the variant PmScsCΔLinker it is a different story; the protein is in equilibrium between different oligomerization states. At 0.1 mg ml^−1^ approximately 97% of the PmScsCΔLinker solution is present as monomers and ∼3% is present as dimers, with negligible trimers. At 10 mg ml^−1^ these proportions change to ∼40% monomers, ∼48% dimers and ∼12% trimers.

## 4. Discussion   

This study focused on characterizing two variants of PmScsC, which in its native form is a highly dynamic, trimeric disulfide isomerase. The native PmScsC protomer consists of an N-terminal trimerization stem with an 11-amino-acid flexible linker connected to a C-terminal thioredoxin-fold catalytic domain harbouring a C*XX*C active site. To better understand the role of the trimerization stem and flexible linker, we designed the variants PmScsCΔN and PmScsCΔLinker. PmScsCΔN lacks the first 41 amino acids of the native PmScsC construct; its loss of disulfide isomerase activity and gain of dithiol oxidase activity has been reported previously (Furlong *et al.*, 2017 ▸). PmScsCΔLinker is newly reported here and lacks the 11 amino acids that form the flexible linker. Here, we have reported the structure of PmScsCΔN and analysed the structure and biochemical function of PmScsCΔLinker.

The X-ray crystal structure of PmScsCΔN agrees with previous MALS data showing that this variant is monomeric (Furlong *et al.*, 2017 ▸). The crystal structure is also consistent with solution SAXS data and the low-resolution model obtained from SAXS analysis. Although the PmScsCΔN crystal structure is very similar to the structures of the catalytic domains in each of the three crystal structures of native PmScsC, unlike the native enzyme it lacks disulfide isomerase (disulfide-shuffling) activity and instead has dithiol oxidase (disulfide-forming) activity. The PmScsC catalytic domain has an acidic catalytic cysteine and an energetically unfavourable disulfide (Furlong *et al.*, 2017 ▸), which are also features of the archetypal but monomeric dithiol oxidase EcDsbA, so it is perhaps not surprising that the removal of the N-terminal trimerization residues leads to oxidase activity in a model assay. Moreover, the structural similarity between monomeric PmScsCΔN and the catalytic domains of native trimeric PmScsC supports the notion that it is the N-terminal stem, which defines whether the catalytic domain is monomeric or trimeric, that is the determinant of isomerase or oxidase activity. Put simply, the differential activity that we observe between native PmScsC and variant PmScsCΔN is not a specific feature of the catalytic domain because the same catalytic domain has the propensity for oxidase (monomeric protein) or isomerase (trimeric protein) activity depending on its context. The isomerase activity of PmScsC requires both its N-terminal trimerization domain and its C-terminal catalytic domain, in the same way that the isomerase activity of EcDsbC requires its dimerization domain and its catalytic domain (Sun & Wang, 2000 ▸).

Our SAXS analysis showed that native PmScsC is a trimer in solution, even at low concentrations. However, SAXS analysis of PmScsCΔLinker showed that it exists predominately as a monomer or a low-affinity dimer (*K*_d_ of ∼200 µ*M*) in preference to a trimer in solution, even though it crystallizes in the trimeric form. Together, these data suggest that removal of the flexible linker of PmScsC between the trimerization stem and catalytic domains has the unintended consequence of reducing the propensity to form a stable trimer in solution.

The crystal structure of PmScsCΔLinker provides insight into why the trimeric form of the variant may be energetically unfavourable compared with the monomeric and dimeric forms in solution. The trimerization stem interactions of PmScsCΔLinker are similar to those observed in the crystal structures of the native enzyme, but all three catalytic motifs are in closer proximity to each other than in any of the native PmScsC crystal structures. This close packing suggests the possibility of unfavourable contacts between catalytic domains in PmScsCΔLinker that may contribute to the instability of the trimer. Indeed, the crystal structure of PmScsCΔLinker reveals a close association of a positively charged patch near the catalytic motif on the accessible surface of one protomer with a neutral/hydrophobic patch on another. In the crystal packing of the PmScsCΔN structure (which does not have the constraint of the trimerization stem), there are no contacts involving the positively charged surface patch near the catalytic motif, suggesting that packing against this site is not favoured. Thus, the formation of the PmScsCΔLinker trimer is likely to be dependent on the balance of favourable interactions formed between the trimerization stems and unfavourable inter­actions between the catalytic domains. At lower concentrations the unfavourable interactions between the catalytic domains may override the favourable interactions, but at higher concentrations, with more molecules in close contact, it may be more thermodynamically favourable for the hydrophobic portions of the trimerization helices to interact with each other. This interplay between interactions of the catalytic domains and trimerization stems could explain why the oligomeric state of PmScsCΔLinker in solution alters with concentration of the protein. Taken together, the crystal structure and SAXS analysis of PmScsCΔLinker suggests that the 11-amino-acid linker plays a dual role by (i) acting as a spacer between the trimerization and catalytic domains to enable the formation of a stable trimer and (ii) providing the necessary functional dynamics and cooperativity between the catalytic domains (Furlong *et al.*, 2017 ▸).

Removal of the 11-amino-acid flexible linker from PmScsC impacts on the activity of the protein: unlike the native protein, PmScsCΔLinker has no disulfide isomerase activity. Structural characterization of PmScsCΔLinker revealed two potential reasons for this result. Firstly, at the concentration used in the assay (10 µ*M*, ∼0.2 mg ml^−1^) it is likely that most of the protein would be monomeric (Fig. 8 ▸ *c*), and we know from PmScsCΔN variant studies that monomeric protein has low protein disulfide isomerase activity (Furlong *et al.*, 2017 ▸). Secondly, even if sufficient trimeric PmScsCΔLinker were present in solution, the crystal structure suggests there would be limited space between the catalytic domains to bind a misfolded protein substrate and possibly limited motion to allow disulfides to be shuffled. It is interesting to note that PmScsCΔLinker is inactive in the dithiol oxidase assay. Given that the protein would be predominantly monomeric at the concentration tested in this assay (80 n*M* or 0.002 mg ml^−1^; Fig. 8 ▸ *c*) we expected that, like monomeric PmScsCΔN, it would have oxidase activity. One explanation for this result is that the N-terminal hydrophobic regions of PmScsCΔLinker somehow occlude the substrate-binding site that is available in monomeric PmScsCΔN.

Overall, our structural and functional characterization of PmScsCΔN and PmScsCΔLinker provides new insight into the importance of unique structural features of the trimeric disulfide isomerase PmScsC. We confirmed previous assumptions that disulfide isomerases are similar to but differentiated from dithiol oxidases by requiring both an oligomerization domain and a catalytic domain. We further propose that the flexible linker of PmScsC is important not just as a shape-shifting peptide, but also as a spacer to enable functional flexibility between the catalytic domains. We suggest that this functional flexibility between the catalytic domains is necessary for the cooperative enzymatic activity of the disulfide isomerase PmScsC. Finally, on a more general note, we found that deleting amino acids from a protein can have unintended consequences. In this case, the deletion of the flexible linker unexpectedly changed the oligomerization kinetics of the protein, even though the region directly involved in trimerization remained intact.

## Supplementary Material

PDB reference: PmScsCΔLinker, 6mhh

PDB reference: PmScsCΔN, 6nen

X-ray diffraction images related to PDB entry 6MHH URL: https://doi.org/10.14264/uql.2018.842

X-ray diffraction images related to PDB entry 6NEN URL: https://doi.org/10.14264/uql.2018.843

## Figures and Tables

**Table 1:** Crystallographic statistics for PmScsCΔN and PmScsCΔLinker Values in parentheses are for the highest resolution shell.

	PmScsCΔN	PmScsCΔLinker
PDB code	6nen	6mhh
Data collection		
Wavelength (Å)	0.9537	1.541870
Resolution (Å)	45.68–2.15 (2.22–2.15)	24.99–2.08 (2.14–2.08)
Space group	*P*312	*H*32/*R*32:*h*
*a*, *b*, *c* (Å)	105.5, 105.5, 35.13	64.04, 64.04, 299.83
α, β, γ (°)	90, 90, 120	90, 90, 120
No. of measured reflections	265709 (21092)	148450 (8858)
No. of unique reflections	12203 (1027)	14715 (1135)
*R*_merge_	0.132 (0.877)	0.059 (0.140)
*R*_meas_	0.135 (0.899)	0.063 (0.150)
Mean *I*/σ(*I*)	21.6 (4.9)	27.2 (11.8)
Multiplicity	21.8 (20.5)	10.1 (7.8)
Completeness (%)	99.7 (97.0)	99.9 (100.0)
Wilson *B* factor (Å^2^)	28.3	16.9
Refinement		
No. of monomers in asymmetric unit	1	1
Resolution used in refinement (Å)	35.13–2.15	24.99–2.08
No. of unique reflections	12196	14704
*R*_free_ (%)	20.8	21.0
*R*_work_ (%)	17.5	17.4
No. of protein atoms (non-H)	1333	1671
No. of ligand atoms	0	0
No. of waters	68	131
*B* factors (Å^2^)		
Average	34.7	30.5
Protein atoms	34.6	30.3
Waters	37.2	33.4
R.m.s.d., bond lengths (Å)	0.004	0.003
R.m.s.d., bond angles (°)	0.815	0.687
*MolProbity* results		
Ramachandran favoured (%)	98.9	99.5
Ramachandran outliers (%)	0.6	0.0
Clashscore	0.37	1.17

**Table 2:** Details of SAXS data collection and analysis

	PmScsCΔN	PmScsC	PmScsCΔLinker
SASBDB ID	SASDEK4	SASDER4	SASDEQ4
Data-collection parameters			
Instrument	SAXS/WAXS, Australian Synchrotron		
Beam geometry	Point		
Wavelength (Å)	1.033		
Sample-to-detector distance (m)	3.330		
*q*-range (Å^−1^)	0.008–0.363		
Temperature (K)	283		
Absolute intensity calibration	Water		
Exposure time (s)	38 (38 × 1 s exposures)		
Configuration	Concentration series from 96-well plate		
Protein concentration range (mg ml^−1^)	0.1–10.0		
Sample details			
Extinction coefficient [*A*_280_, 0.1%(*w*/*v*)]	0.643	0.528	0.558
Partial specific volume (cm^3^ g^−1^)	0.742	0.741	0.743
Contrast, Δρ (10^10^ cm^−2^)	2.79	2.80	2.78
Molecular mass (from sequence) (kDa)	20.2	24.8	23.4
Protein concentration (mg ml^−1^)	0.50†	0.1, 0.5, 2.0, 5.0, 10.0	0.1, 0.5, 2.0, 5.0, 10.0
Structural parameters			
*I*(0) (from Guinier) (cm^−1^)	0.004055 ± 0.00001	—	—
*R*_g_ (from Guinier) (Å)	17.0 ± 0.1	—	—
*I*(0) [from *p*(*r*)] (cm^−1^)	0.004053 ± 0.00001	—	—
*R*_g_ [from *p*(*r*)] (Å)	17.0 ± 0.1	—	—
*D*_max_ (Å)	55 ± 2	—	—
Porod volume (Å^3^)	22200 ± 1000	—	—
Molecular-mass determination			
Molecular mass [from *I*(0)] (kDa)	11 ± 2	—	—
Molecular mass (from Porod) (kDa)	19 ± 1	—	—

† PmScsCΔN: *I*(0) = 0.00140 ± 0.00002 cm^−1^, *R*_g_ = 16.9 ± 0.3 Å, *M* = 19.8 kDa (0.1 mg ml^−1^); *I*(0) = 0.004053 ± 0.000001 cm^−1^, *R*_g_ = 17.0 ± 0.1 Å, *M* = 19.1 kDa (0.5 mg ml^−1^); *I*(0) = 0.01839 ± 0.00003 cm^−1^, *R*_g_ = 17.1 ± 0.1 Å, *M* = 19.3 kDa (2.0 mg ml^−1^); *I*(0) = 0.05463 ± 0.00005 cm^−1^, *R*_g_ = 17.2 ± 0.1 Å, *M* = 19.6 kDa (5.0 mg ml^−1^); *I*(0) = 0.10756 ± 0.00005 cm^−1^, *R*_g_ = 17.0 ± 0.1 Å, *M* = 19.2 kDa (10.0 mg ml^−1^). Masses from the Porod volume are used as concentration estimates, yielding masses from *I*(0) inconsistent with the expected mass. The 0.5 mg ml^−1^ data were used for subsequent data analysis.

**Table 3:** Oligomer fractions and equilibrium constants for dimer and trimer formation (*K*_d_ and *K*_t_, respectively) from analysis of the SAXS data Values obtained from the global optimization are shown first and those from the independent optimization are given in parentheses.

Measured concentration (mg ml^−1^)	Calculated concentration (mg ml^−1^)	Monomer fraction	Dimer fraction	Trimer fraction
Native PmScsC (*K*_t_ = 6.3 × 10^−18^ *M* ^2^)				
0.1	0.05 (0.06)	0.01 (0.21)	—	0.99 (0.79)
0.5	0.33 (0.33)	0.00 (0.04)	—	1.00 (0.96)
2.0	1.48 (1.48)	0.00 (0.00)	—	1.00 (1.00)
5.0	3.74 (3.74)	0.00 (0.00)	—	1.00 (1.00)
10.0	7.07 (7.08)	0.00 (0.00)	—	1.00 (1.00)
PmScsCΔLinker (*K*_d_ = 2.1 × 10^−4^ *M*; *K*_t_ = 7.3 × 10^−4^ *M*)				
0.1	0.07 (0.07)	0.97 (1.00)	0.03 (0.00)	0.00 (0.00)
0.5	0.36 (0.36)	0.88 (0.94)	0.12 (0.00)	0.00 (0.06)
2.0	1.31 (1.30)	0.71 (0.67)	0.27 (0.33)	0.02 (0.00)
5.0	3.55 (3.55)	0.53 (0.55)	0.41 (0.37)	0.07 (0.08)
10.0	7.17 (7.15)	0.40 (0.37)	0.48 (0.49)	0.12 (0.10)
